# Rapid Microsatellite Marker Development Using Next Generation Pyrosequencing to Inform Invasive Burmese Python—*Python molurus bivittatus*—Management

**DOI:** 10.3390/ijms14034793

**Published:** 2013-02-27

**Authors:** Margaret E. Hunter, Kristen M. Hart

**Affiliations:** 1 U.S. Geological Survey, Southeast Ecological Science Center, 7920 NW 71st Street, Gainesville, FL 32653, USA; 2 U.S. Geological Survey, Southeast Ecological Science Center, 3205 College Avenue, Davie, FL 33314, USA; E-Mail: kristen_hart@usgs.gov

**Keywords:** invasive species, exotic species, next generation sequencing, microsatellite marker, Everglades National Park, Roche 454, cross-species amplification

## Abstract

Invasive species represent an increasing threat to native ecosystems, harming indigenous taxa through predation, habitat modification, cross-species hybridization and alteration of ecosystem processes. Additionally, high economic costs are associated with environmental damage, restoration and control measures. The Burmese python, *Python molurus bivittatus*, is one of the most notable invasive species in the US, due to the threat it poses to imperiled species and the Greater Everglades ecosystem. To address population structure and relatedness, next generation sequencing was used to rapidly produce species-specific microsatellite loci. The Roche 454 GS-FLX Titanium platform provided 6616 di-, tri- and tetra-nucleotide repeats in 117,516 sequences. Using stringent criteria, 24 of 26 selected tri- and tetra-nucleotide loci were polymerase chain reaction (PCR) amplified and 18 were polymorphic. An additional six cross-species loci were amplified, and the resulting 24 loci were incorporated into eight PCR multiplexes. Multi-locus genotypes yielded an average of 61% (39%–77%) heterozygosity and 3.7 (2–6) alleles per locus. Population-level studies using the developed microsatellites will track the invasion front and monitor population-suppression dynamics. Additionally, cross-species amplification was detected in the invasive Ball, *P. regius*, and Northern African python, *P. sebae*. These markers can be used to address the hybridization potential of Burmese pythons and the larger, more aggressive *P. sebae*.

## 1. Introduction

As the number of invasive organisms steadily increases, the development and implementation of effective management actions will require knowledge of discrete breeding populations, dispersal patterns and fluctuations in population size. Operational protocols, methods and tools could improve these rapid-response control and eradiation actions. One such tool, next generation sequencing (NGS), can rapidly and cost-effectively generate microsatellite markers to help track invasion dynamics and pathways, determine the genetic relationships of native and invasive populations and improve rapid-response containment or removal efforts [1–3]. Further, microsatellite markers can be used to reconstruct pathways of introduction by assessing the relationship of invasive populations throughout the landscape.

DNA sequencing represents an efficient and cost-effective method for identifying large numbers of microsatellites, especially in non-model organisms with limited genomic information. Traditional, cloning-based hybrid-capture microsatellite development using Sanger sequencing is time-consuming, technically demanding and considerably costlier [4,5], whereas NGS of genomic DNA can rapidly and cost-effectively identify up to an order of magnitude more microsatellite sequences [4,6–8]. Typically, NGS protocols sequence from one to five individuals [9] on one or two flow cell lanes using genomic shotgun libraries [8], microsatellite enriched libraries [1,4] or transcriptome sequences [10]. Molecular Identifier (MID) tags, also known as barcodes, can be included on the sequencing adapters to differentiate multiple samples in a single lane [7,11].

Non-targeted and unassembled reads of genomic shotgun libraries have emerged as the most efficient and economical strategy for detecting microsatellites in non-model organisms [11–13]. Targeted enrichment and hybridization protocols use *a priori* motif selection to target microsatellite repeats, which can increase the discovery rate, but also limits the available types of repeat motifs [1]. Further, the implementation of previously developed markers from closely related species has proven useful; however, it has been shown to have lower success rates as evolutionary distance increases [14,15].

In order to develop highly informative molecular markers, Roche 454 pyrosequencing was used to rapidly generate low-cost, long-read and species-specific microsatellites to inform management of the invasive Burmese python, *Python molurus bivittatus*, also classified as *Python bivittatus*[16]. The number of *P. m. bivittatus* individuals in Everglades National Park (ENP), FL, USA, has been estimated to be greater than 30,000 [17,18], although fine-scale population structure, level of effective movement and differential dispersal have not been determined. A previous *P. m. bivittatus* genetic report indicated a single breeding population within ENP, with no variation at cytochrome *b* and low variation at cross-species microsatellite markers [19]. However, a direct comparison cannot be made, because full details were not reported, including some identifying primer information [19]. Recently, potentially amplifiable loci (PALs) were identified in the *P. m. bivittatus* using Illumina paired-end sequences; however, no microsatellite loci were tested for amplification or polymorphism [12].

*Python molurus bivittatus* represents a highly injurious invasive species to the Florida Everglades ecosystem, reaching lengths of 5.4 m and consuming mammals, birds and reptiles [20,21]. Strong population-level declines or near disappearances have been documented for many mammal species where *P. m. bivittatus* has become established [22]. This disruption to the Everglades trophic structure will likely have severe impacts on the south Florida ecosystem as a whole. Furthermore, many native and imperiled species are at risk of population decline or loss due to python predation. Through gut content studies, the Federally endangered Key Largo woodrat, *Neotoma floridana smalli*, threatened American alligator, *Alligator mississippiensis*, and species of special concern, such as the limpkin, *Aramus guarauna*, and white ibis, *Endocemus albus*, have been identified as python prey species [23]. In addition, Burmese pythons are believed to be interspecific competitors with the federally threatened indigo snake, *Drymarchon couperi*, already imperiled by habitat loss and fragmentation [24].

## 2. Results and Discussion

The Roche 454 pyrosequencing run using Titanium chemistry yielded 32,693,771 base pairs of data in 117,516 reads with an average read length of 278 base pairs and an average quality score of 29.3. Msatcommander 0.8.2 [25] identified 6616 microsatellite repeats, of which 1313 (19.8%) were suitable for primer design (Table 1). Of those loci, 64 were composed of compound repeats, while 142 had interrupted and compound repeats. Dinucleotide microsatellites with ≥10 repeated motifs were contained in the most sequences. While, within sequences suitable for primer design, tetranucleotide microsatellites with ≥10 repeats were contained in the largest number of loci (Table 1).

Only perfect tri- or tetra-nucleotide repeat motifs with 7–21 repeat copies were selected for primer design. Of the 26 loci that were selected (NCBI Sequence Read Archive accession numbers SRS387505, 06, 31–54), eight were removed from the analyses; two did not amplify, two could not be easily genotyped and four were monomorphic. The suitable 18 species-specific loci (NCBI PUID 16822100-17) were combined with the six cross-species microsatellites [26] to create eight multiplexes with 1–4 primers (Tables 2 and 3). Over the 24 loci, allelic diversity ranged from 2 to 6 alleles per locus with an average of 3.7 in 20 snakes. Expected heterozygosities ranged from 39% to 77%, with an average of 61%. For the 18 species-specific and six cross-species loci, the allelic diversity averaged 3.4 and 4.5, and the heterozygosity averaged 61% and 63%, respectively.

The markers provided enough power to produce unique multilocus genotypes for accurate identification of individuals within the population. The probability of identity (*P*_ID_) indicated a low probability that two siblings (*P*_(ID)sib_ = 8.6 × 10^−8^) or two randomly selected individuals (*P*_ID_ = 3.1 × 10^−16^) would have identical genotypes. Over 276 comparisons, only a single pair-wise comparison (*Pmb-U21* and *Pmb-*O15) indicated linkage disequilibrium after a sequential Bonferroni adjustment (overall α = 0.05, *p* = 0). Only *Pmb-U21* (α = 0.01, *p* ≤ 0.0001) showed signs of null alleles, due to an excess of homozygotes, and deviated from expectations after Hardy-Weinberg equilibrium (HWE) tests for conformation. No duplicate genotypes were found in the data set, all individuals differed at 14 or more loci. A subset of the loci was identified as amplifiable and polymorphic in small sample sizes of invasive North African pythons, *P. sebae*, and Ball pythons, *P. regius*, at the *P. m. bivittatus* PCR conditions (Tables 2 and 3).

Rapid tools and techniques are needed to assist resource managers in understanding population dynamics for control and eradication of invasive species. The developed *P. m. bivittatus* microsatellites can be used for phylogenetics, landscape genetics, kinship, relatedness, bottleneck, individual identification and effective population size studies in invasive south Florida populations. Further, these markers could improve conservation genetic studies in the native range of *P. m. bivittatus*, where the species is considered vulnerable by the International Union for Conservation of Nature Red List [27]. The longer read-lengths provided by Titanium chemistry and the ability to select only perfect repeats allow for more microsatellite loci to be identified with an adequate primer sequence and more repeats within primers to increase the statistical power of the microsatellites [28,29]. Discovery rates for identifying suitable microsatellites in unassembled NGS data can vary widely (25%–77%), depending on the species, enrichment procedure and sequence quality [7,9,30]. Of the screened primers in this study, 69% reliably amplified and were polymorphic. In previous cross-species studies, 10 of 27 loci (37%) were suitable for population genetic analyses, although little genetic differentiation was identified in ENP Burmese python samples [19].

## 3. Experimental Section

### 3.1. Sample Preparation and 454 Pyrosequencing

Genomic DNA was isolated from *P. m. bivittatus* and *P. sebae* specimens collected in ENP and fresh *P. regius* skin sheds from a personal collection. All animals used in the project were engaged, collected and handled in a manner consistent with the National Park Service IACUC guidelines and mandates. DNA from frozen muscle tissue and shed skins was isolated following Qiagen’s DNeasy Blood and Tissue kit (Valencia, CA, USA) protocol. DNA quality was assessed using spectrophotometric absorbance and electrophoresis on a 1% agarose gel. DNA prepared for the NGS shotgun library was pooled from four *P. m. bivittatus* (two male and two female; 162 μg) for the sequencing of 1/8 plate on the Roche 454 Genome Sequencer FLX Titanium platform (Interdisciplinary Center for Biotechnology Research, University of Florida, Gainesville, FL, USA). Library construction and sequencing followed Roche 454 standard protocols. Briefly, DNA was fragmented into 500–800 bp pieces and ligated onto Rapid Library Adaptors. The fragments were amplified on beads through emulsion PCR and sequenced using the standard GS-FLX Titanium reagents. The Roche 454 Genetic Sequencing Analysis Software package was used for raw read image and quality processing.

### 3.2. Marker Selection

The program Msatcommander 0.8.2 [25] was used to search for microsatellite repeats within the NGS data. Screening was conducted on perfect di-, tri- and tetra-nucleotide repeats with a minimum of six repeats each. Primers were designed for suitable singleton sequences in the interfacing Primer 3 software package [31]. Primer criteria included a GC content of 40%–60%, final product length of 150–350 base pairs, optimal annealing temperature of 60 °C, a location of 10 base pairs from start or end positions and maximum homopolymer of four nucleotides within the primer sequence. Microsatellite loci with compound (two or more types of repeats) or compound and interrupted repeats were excluded from primer selection and optimization.

### 3.3. Marker Optimization

From the suitable sequences, 26 primers were randomly selected for PCR amplification and screening. To enable rapid primer testing, an M13 sequence tag was added to the 5′ of the forward primer [32], complementary to a third primer, which was 5′-fluorescently labeled with 6-FAM or HEX. Primers were initially screened on eight individuals in 20 μL PCR reactions: 10 ng DNA, 1× PCR buffer (10 mM Tris-HCl, pH 8.3, 50 mM KCl, 0.001% gelatin; Sigma-Aldrich, Inc., St. Louis, MO, USA), 0.8 mM dNTP, 2 mM MgCl_2_, 0.1 μM M13 primer, 0.25 μM long primer, 0.5 μM short primer, 0.05 units of Sigma Jump Start *Taq*DNA polymerase. The PCR cycling profile was: 5 min at 95 °C; then 35 cycles of 30 s at 95 °C, 1 min at T_a_ (Table 2), 2 min at 70 °C; then 10 min at 72 °C. Amplified products were analyzed on an Applied Biosystems 3130xl and scored with GeneMarker (SoftGenetics, LLC, State College, PA, USA). Annealing temperatures were adjusted accordingly for poor or non-specific amplification. Loci resulting in inconsistent amplification or performance were subsequently excluded from further testing. The selected polymorphic loci were tested on a total of 20 samples located throughout the Everglades ecosystem (Table S1) to estimate genetic diversity parameters. Two related and exotic species also found in ENP, *P. sebae* (*n* = 2) and *P. regius* (*n* = 3), were tested with the developed multiplexes for amplification and polymorphism.

Collins *et al.*[19] tested the cross-species reactivity and diversity of 27 *Morelia spilota* microsatellites in *P. m. bivittatus* samples from ENP and Vietnam [26]. Of the loci tested, 10 were identified as unambiguous and polymorphic, although some locus names were not presented. Additionally, individual locus genetic diversity values were not reported for the species, and some average and overall values were not reported for the ENP python population separately. From the 10 cross-species loci utilized, six were optimized for use in this study. PCR multiplexes were created with the species-specific and cross-species primers using Multiplex Manager[33]. The primers were directly labeled with 6-FAM, HEX or NED fluorescent dye for use in the multiplexes (Tables 2 and 3).

GenAlEx 6.4 [34] was used to calculate the average number of alleles and observed and expected heterozygosity. Genepop 4.0.10 [35] assessed deviations from Hardy–Weinberg equilibrium (HWE) and linkage disequilibrium between pairs of loci. Micro-Checker v. 2.2.3 [36] tested for evidence of null alleles, large allelic dropout and genotyping errors. Genecap[37] calculated the unbiased probability of identity (*P*_ID_), which is the probability that two individuals drawn at random from a population will have the same genotype at the assessed loci [38], and *P*_(ID)sib_, a related, more conservative statistic for calculating *P*_ID_ among siblings or related individuals [39]. The program additionally searched for duplicate genotypes among different individuals.

## 4. Conclusions

These markers will assist with identifying detailed population structure across the ENP, the level of effective movement and differential dispersal rates within the south Florida and Florida Keys Burmese python populations. Further, the nuclear microsatellites will be useful to detect hybrid crosses between Burmese pythons and the larger and more aggressive Northern African pythons, as has been hypothesized. In this study, enough statistical power (*P*_ID_ = 3.1 × 10^−16^) was achieved to confidently identify unique individuals in a population with approximately 3.2 × 10^15^ snakes, more than estimated in the Everglades population. This high degree of power is necessary given the previously determined lack of mitochondrial sequence diversity and nuclear population structure [19]. The diversity may be reduced due to founding events in the pet-trade, a likely source of the invasive Burmese python population [18].

Additional samples will be investigated to generate more robust population-level and range-wide diversity and genetic structure analyses. The rapid development of microsatellite markers will help to track the dynamics of the invasion and containment or removal methods. The developed molecular tools will provide critical information for decision-making processes, as various species in the *Python* genus continue to expand their non-native range and alter the Greater Everglades ecosystem.

## Supplementary Information



## Figures and Tables

**Table 1:** Numbers and types of microsatellites identified in 454 GS-FLX Titanium reads from *Python molurus bivittatus* genomic DNA. Microsatellite loci containing compound and compound/interrupted repeat units were excluded from the primer design.

Repeat motif	Repeats/locus	Sequenced loci	Loci with designed primers	Compound loci	Compound/Interrupted loci
Dinucleotide	All (≥6)	2411	423	8	38
	≥10	868	55	-	7
	≥20	70	-	-	-
Trinucleotide	All (≥4)	2134	334	29	48
	≥10	341	12	11	4
	≥20	12	-	0	-
Tetranucleotide	All (≥4)	2071	447	27	56
	≥10	624	90	8	11
	≥20	20	4	-	-

**Table 2:** Characteristics of polymorphic microsatellite loci developed in invasive *Python molurus bivittatus*. All loci were amplified by 20 *P. m. bivitattus* samples. Cross-species amplification was tested on additional invasive species: the Northern African python (*P. sebae; n* = 2) and Ball python (*P. regius; n* = 3). Locus designation, repeat motif and number of repeats, primer sequence (5′→3′), 5′ primer fluorescent dye, allele size range, polymerase chain reaction (PCR) multiplex (MP) of each locus, annealing temperature (Ta), average number of alleles (Na), effective number of alleles (Ne), observed heterozygosity (Ho), expected heterozygosity (He) and number of alleles (above) and average size of PCR products (below) in *P. sebae* and *P. regius.* X indicates that the locus was not resolvable under the PCR conditions developed for *P. m. bivitattus*.

Locus	Repeat motif	Primer sequence (5′-3′)	Dye	Allele range	MP	Ta	Na	Ne	Ho	He	*P. sebae*	*P. regius*
*Pmb-A01*	(CCT)_7_	F: AAGCTGCTGATGTCCAGGC	6-FAM	246–249	MP4	59	2	1.98	0.50	0.51	1	X
		R: ATGGCTATCTCCGCTGTCC									246	
*Pmb-B02*	(ACAT)_8_	F: GGAGTTCTGTTCTACAGGTGC	HEX	234–242	MP2	61	3	2.74	0.55	0.65	X	X
		R: TGTGCCTTCAAATCCAGCG										
*Pmb-D04*	(CATT)_12_	F: TCATCAACCTGAGCCAACAG	6-FAM	307–323	MP2	61	4	2.84	0.60	0.66	1	2
		R: GAGCAATTGGGAGTCAGGC									312	258
*Pmb-F06*	(ACAT)_11_	F: TCAAACTCTCAGGCCTCTGG	HEX	266–274	MP3	62	2	1.60	0.40	0.38	2	X
		R: ATAGGGTCCATGGGAGCAG									263	
*Pmb-G07*	(GAT)_15_	F: TCTCTGGAATCAGGCAGAACC	HEX	169–183	MP8	62	3	2.66	0.60	0.64	X	X
		R: ATCCCTCCAGACACACACC										
*Pmb-J10*	(ATGT)_10_	F: TCCTCGGCTGACTTCCTTG	6-FAM	305–309	MP5	60	2	1.96	0.55	0.50	1	X
		R: TCCATCTAACGACCCTTGC									314	
*Pmb-K11*	(AGAT)_14_	F: TTTGCTGCCCAGAGTTGTC	FAM	194–202	MP6	57	3	2.87	0.65	0.67	3	2
		R: AGCAGTTTGACCTCATTCCAG									186	182
*Pmb-L12*	(ATC)_12_	F: GCCACGTCTAAGGTTGAGC	HEX	157–169	MP6	57	4	2.37	0.55	0.59	2	2
		R: AAAGCAGGTCTCTGTTGGG									146	142
*Pmb-N14*	(GAT)_13_	F: TTGGTAGTGGTGGTGGTGG	6-FAM	207–216	MP3	62	4	3.04	0.60	0.69	3	2
		R: GGCTGGCTGCTACTGAAAC									148	188
*Pmb-O15*	(AATC)_7_	F: TAGAGGGCAGTTTGGACCC	6-FAM	184–196	MP2	61	3	2.82	0.60	0.66	X	3
		R: ATGGGCACACTTTGAAGCC										190
*Pmb-Q17*	(AAAT)_11_	F: CTGTTCTACCTGACAACTTCCC	HEX	154–182	MP3	62	5	4.02	0.80	0.77	2	1
		R: TCTAGCCCAAGTGACAGGAAC									106	163
*Pmb-R18*	(ATTT)_10_	F: AGCAGCCCACGTAGAGTATG	6-FAM	189–221	MP5	60	4	2.86	0.65	0.67	X	2
		R: GGTCACCAAGATGGTTGGG										187
*Pmb-S19*	(ATTT)_11_	F: AGCTAGTAAGCATAGGGAAGGC	NED	160–172	MP2	61	3	1.87	0.60	0.48	X	X
		R: TCCTTTGTTGAAATGGGTGGC										
*Pmb-T20*	(AGG)_8_	F: GGGTTCGCTACTTTTCCGC	6-FAM	226–233	MP8	62	3	1.70	0.35	0.42	X	X
		R: TTCGCCTCACCCTTTCTGG										
*Pmb-U21*	(AATG)_11_	F: GGAGTTTAGCGAAGTTGGGC	6-FAM	252–285	MP2	60	4	2.64	.025	0.64	X	3
		R: CAGTCTAAGCTATGACCTTGGG										190
*Pmb-V22*	(ATTT)_7_	F: TCGGATGTGGCACTGAAGG	HEX	233–253	MP5	60	4	2.12	0.60	0.54	1	3
		R: GCCCAATGTGTGACAAGGC									220	184
*Pmb-W23*	(AATG)_13_	F: AGCCACAATAAGCAATGTAGGTC	NED	156–168	MP4	59	4	3.94	0.75	0.77	1	2
		R: CCAAGTTACACTCTTCCATGTTCC									170	142
*Pmb-Z26*	(CATT)_10_	F: ATGCCAAGGTATCAGGGCTC	NED	148–160	MP5	60	4	2.81	0.55	0.66	1	3
		R: AGCTAGAGCTCAATTCTCCAG									150	143

**Table 3:** Cross-species characterization of 20 *Python molurus bivittatus* samples amplified by *Morelia spilota* microsatellite primers [26]. Amplification and polymorphism was also tested on the Northern African python (*P. sebae; n* = 2) and Ball python (*P. regius; n* = 3). Locus designation, 5′ primer fluorescent dye, allele size range, PCR multiplex (MP) of each locus, annealing temperature (Ta), average number of alleles (Na), effective number of alleles (Ne), observed heterozygosity (Ho), expected heterozygosity (He) and number of alleles and average size of PCR products in *P. sebae* and *P. regius.* X indicates that the locus was not resolvable under the PCR conditions developed for *P. m. bivitattus*.

Locus	Dye	Allele range	MP	Ta	Na	Ne	Ho	He	*P. sebae*		*P. regius*	

									Na	Allele size	Na	Allele size
*MS9*	6-FAM	177–194	MP1	54	5	2.27	0.60	0.57	2	168	3	172
*MS10*	HEX	218–238	MP4	59	5	2.08	0.60	0.53	1	249	3	189
*MS11*	HEX	344–400	MP7	57	6	2.80	0.85	0.66	X	-	3	447
*MS13*	HEX	176–198	MP7	57	3	2.53	0.55	0.62	X	-	4	243
*MS16*	6-FAM	355–383	MP6	57	4	3.14	0.75	0.70	1	343	3	351
*MS22*	6-FAM	394–423	MP4	59	4	3.07	0.60	0.69	X	-	2	349
