# Supplementary material for: Rapid Microsatellite Marker Development Using Next Generation Pyrosequencing to Inform Invasive Burmese Python—Python molurus bivittatus—Management
Source: Int J Mol Sci. 2013 Feb 27;14(3):4793–804. doi: 10.3390/ijms14034793 (PMC3634432; doi:10.3390/ijms14034793)
Supplement: Supplementary file 1 [file ijms-14-04793-s001.docx]

Supplementary Information

**Table S1.** Sample name, Universal Transverse Mercator (UTM) coordinate, East and North, collection date, and arbitrary Greater Everglades ecosystem collection zone.

| **Sample** | **UTM-East** | **UTM-North** | **Collection date** | **Collection Zone** |
| --- | --- | --- | --- | --- |
| PMB-1 | 562384 | 2815279 | 1-Jan-10 | 2 |
| PMB-2 | 519060 | 2849339 | 5-Jan-10 | 1 |
| PMB-3 | 523435 | 2849350 | 5-Jan-10 | 1 |
| PMB-4 | 530446 | 2849356 | 5-Jan-10 | 1 |
| PMB-5 | 524307 | 2849358 | 5-Jan-10 | 1 |
| PMB-6 | N/A | N/A | 2-Jan-10 | 1 |
| PMB-7 | 555789 | 2797171 | 6-Jan-10 | 2 |
| PMB-8 | 554252 | 2773410 | 6-Jan-10 | 3 |
| PMB-9 | 550316 | 2848661 | 7-Jan-10 | 5 |
| PMB-10 | 552178 | 2845821 | 13-Jan-10 | 5 |
| PMB-11 | 514503 | 2853159 | 28-Jan-10 | 1 |
| PMB-12 | 553708 | 2806547 | 8-Feb-10 | 1 |
| PMB-13 | 560036 | 2779974 | 6-Feb-10 | 3 |
| PMB-14 | 547356 | 2830401 | 18-Feb-10 | 5 |
| PMB-15 | 514438 | 2852750 | 23-Feb-10 | 1 |
| PMB-16 | N/A | N/A | 19-Feb-10 | 1 |
| PMB-17 | N/A | N/A | 4-Mar-10 | 1 |
| PMB-18 | N/A | N/A | 7-Mar-10 | 1 |
| PMB-19 | 465912 | 2864928 | 15-Mar-10 | 1 |
| PMB-20 | 428538 | 2871461 | 11-Mar-10 | 1 |

© 2013 by the U.S. Government; licensee MDPI, Basel, Switzerland. This article is an open access article distributed under the terms and conditions of the Creative Commons Attribution license (http://creativecommons.org/licenses/by/3.0/).
